# HGF Gene Modification in Mesenchymal Stem Cells Reduces Radiation-Induced Intestinal Injury by Modulating Immunity

**DOI:** 10.1371/journal.pone.0124420

**Published:** 2015-05-01

**Authors:** Hua Wang, Rui-Ting Sun, Yang Li, Yue-Feng Yang, Feng-Jun Xiao, Yi-Kun Zhang, Shao-Xia Wang, Hui-Yan Sun, Qun-Wei Zhang, Chu-Tse Wu, Li-Sheng Wang

**Affiliations:** 1 Department of Experimental Hematology, Beijing Institute of Radiation Medicine, Beijing, 100850, PR China; 2 College of Life Science and Bioengineering, Beijing University of Technology, Beijing, 100022, PR China; 3 Department of Experimental Pathology, Beijing Institute of Radiation Medicine, Beijing, 100850, PR China; 4 Collaborative Innovation Center for Biotherapy, West China Hospital, Sichuan University，Chengdu, 610041, PR China; University of Cincinnati, College of Medicine, UNITED STATES

## Abstract

**Background:**

Effective therapeutic strategies to address intestinal complications after radiation exposure are currently lacking. Mesenchymal stem cells (MSCs), which display the ability to repair the injured intestine, have been considered as delivery vehicles for repair genes. In this study, we evaluated the therapeutic effect of hepatocyte growth factor (HGF)-gene-modified MSCs on radiation-induced intestinal injury (RIII).

**Methods:**

Female 6- to 8-week-old mice were radiated locally at the abdomen with a single 13-Gy dose of radiation and then treated with saline control, Ad-HGF or Ad-Null-modified MSCs therapy. The transient engraftment of human MSCs was detected via real-time PCR and immunostaining. The therapeutic effects of non- and HGF-modified MSCs were evaluated via FACS to determine the lymphocyte immunophenotypes; via ELISA to measure cytokine expression; via immunostaining to determine tight junction protein expression; via PCNA staining to examine intestinal epithelial cell proliferation; and via TUNEL staining to detect intestinal epithelial cell apoptosis.

**Results:**

The histopathological recovery of the radiation-injured intestine was significantly enhanced following non- or HGF-modified MSCs treatment. Importantly, the radiation-induced immunophenotypic disorders of the mesenteric lymph nodes and Peyer’s patches were attenuated in both MSCs-treated groups. Treatment with HGF-modified MSCs reduced the expression and secretion of inflammatory cytokines, including tumor necrosis factor alpha (TNF-α) and interferon-gamma (IFN-γ), increased the expression of the anti-inflammatory cytokine IL-10 and the tight junction protein ZO-1, and promoted the proliferation and reduced the apoptosis of intestinal epithelial cells.

**Conclusions:**

Treatment of RIII with HGF-gene-modified MSCs reduces local inflammation and promotes the recovery of small intestinal histopathology in a mouse model. These findings might provide an effective therapeutic strategy for RIII.

## Introduction

Radiation exposure may occur due to a nuclear accident or radiotherapy. The development of novel approaches that protect living tissues from radiation-induced damage is an important field in radiation biology. Intestinal mucosa injury is the major determinant of survival in patients exposed to high doses of radiation [1]. However, there is currently no effective treatment against radiation-induced intestinal injury (RIII) [2].

Mesenchymal stem cells (MSCs) are pluripotent progenitor cells that contribute to the maintenance and regeneration of various connective tissues, including bone, adipose, cartilage and muscle tissue. MSCs are currently among the most advanced cell therapy tools, and many approved MSC products are available, such as Prochymal, Provacel, Chondrogen, Cuepistem, Cartistem and Hearticellgram-AMI [3–6]. Several experimental and clinical studies have reported the therapeutic effects of MSCs in alleviating gastrointestinal disorders in patients with severe steroid-resistant graft-versus-host disease (GVHD)， and the recovery of rectovaginal and perianal fistulas in patients with Crohn’s disease [7, 8]. Immunomodulatory activities, such as the suppression of T-cell proliferation and the distribution of MSCs in inflamed tissue, are considered as the central mechanisms of action of MSCs [9, 10].

Hepatocyte growth factor (HGF), which was originally identified and cloned as a potent mitogen in mature hepatocytes, displays mitogenic, morphogenic, and anti-apoptotic activities in a wide variety of cells, preferentially in most epithelial and endothelial cell types. HGF enhances the regeneration of organs such as the intestine, the liver, the kidney and the lung by modulating epithelial cell proliferation and migration [11, 12]. The recombinant HGF protein exerts a therapeutic effect on several animal models of inflammatory bowel disease (IBD) by modulating intestinal epithelial cell proliferation, migration and inflammation [13, 14]. However, the systemic administration of rh-HGF for the treatment of intestinal injury is limited due to the adverse effects caused by high serum HGF concentrations [12].

MSCs exhibit a natural tropism toward injured tissues, and this property delivers repair genes to radiation-injured intestinal tissue. In this study, we evaluated the therapeutic effects of MSC-mediated HGF gene expression on RIII using an *in vivo* C57BL/6 mouse model and elucidated the mechanisms underlying these effects.

## Materials and Methods

### Isolation, culture and characteristics of umbilical cord-derived MSCs

Human umbilical cords were obtained from the *Beijing Hospital of Chinese Traditional and Western Medicine* with the written informed consent of parturients. Umbilical cord-derived MSCs (UC-MSCs) were isolated as described previously with minor modifications [15]. The Ethics Committee of the Beijing Institute of Radiation Medicine approved all of the experiments. Briefly, after the blood vessels were removed, Wharton’s jelly was minced into 1–2 mm^3^ fragments, suspended in an animal serum-free MSC growth medium and incubated in a humidified atmosphere containing 5% CO_2_ at 37°C. The medium was changed every 4 days, and the tissue fragments were removed after 10 days. Once 80% confluence had been reached, the cells were harvested using 0.05% trypsin-EDTA and re-seeded in new flasks at a density of 5×10^4^ cells/ml. Cells at passage 3 were used for experimentation.

The immunophenotypes of MSCs were detected via flow cytometry (BD Biosciences, San Jose, CA), which showed that the MSCs were positive for CD73, CD90 and CD105 and negative for CD34, CD45, and HLA-DR. The adipocytic and osteogenic differentiation capacities of UC-MSCs were also characterized (data not shown).

### Adenoviral vectors (AdVs)

The AdVs used in this study were as follows: Ad-HGF, a replication-defective adenovirus expressing human HGF (hHGF), and Ad-Null, a replication-defective adenovirus not carrying any exogenous genes. All AdVs were constructed using the AdEasy System (Stratagene, La Jolla, CA). The AdVs were purified via double CsCl density gradient ultracentrifugation, dissolved in storage buffer (Hank’s buffer containing 10% glycerol) and stored at -80°C. Viral particle (vp) numbers and infectious titers (infectious units (IU) per ml) were determined as previously described [16], the multiplicity of infection (MOI) was calculated from the infectious titers.

UC-MSCs were infected with 150 MOI of Ad-HGF (MSCs-HGF) or Ad-Null (MSCs-Null), and the cells were collected 48 h post-infection for RIII therapy.

### Animal care

C57BL/6 mice (6–8 wk old, 18–20 g) were obtained from the Academy of Military Medical Sciences (Beijing, China) and were housed and cared for in a pathogen-free facility. All animal experiments were performed in accordance with the Guide for the Care and Use of Laboratory Animals and were approved by the Ethics Committee of the Beijing Institute of Radiation Medicine.

One hundred and twenty mice received a single 13-Gy dose of radiation that was administered locally to the abdomen using gamma rays. Then, the mice were randomized into three groups and injected intravenously via the tail vein 6 h post-radiation with saline as a control (100 μl), MSCs-Null (1×10^6^ cells/100 μl saline) or MSCs-HGF (1×10^6^ cells/100 μl saline). The mice were sacrificed at day 1, 7, 14 or 28 after treatment. Serum was collected for the determination of cytokine levels. The mesenteric lymph nodes (MLNs) and Peyer's patches (PPs) were collected to analyze the phenotype of the lymphocytes. The intestine was removed for RNA or genomic DNA isolation and histopathological analysis.

### Immunophenotypic characterization of the lymphocytes from the MLN and PP

Small intestines were collected, and PPs were carefully removed. The MLNs and PPs were mechanically dissociated in phosphate-buffered saline (PBS) containing 3% fetal calf serum. Lymphocytes were isolated from the MLNs and PPs and were labeled with the mouse regulatory T cell staining kit (clone FJK-16s), anti-mouse CD3 FITC (clone 17A2), anti-mouse CD19 PerCP-Cy5.5 (clone eBio 1D3), anti-mouse CD4 PE (clone GK1.5), anti-mouse CD8b APC (clone eBioH35-17.2) or an isotype control (eBioscience, San Diego, CA). The stained cells were analyzed using a flow cytometer (Becton Dickinson, Mountain View, CA, USA) and Cellquest software.

### Detection of cytokine expression via enzyme-linked immunosorbent assay (ELISA) and real-time polymerase chain reaction (PCR)

Blood samples were obtained from the tail vein of the mice at days 1, 7, 14 and 28 post-radiation. The concentrations of cytokines (TNF-a, IFN-γ and IL-10) were measured using murine cytokine-specific Quantikine ELISA kits (R&D Systems, Minneapolis, MN) according to the manufacturer's instructions. The optical density (OD) value was measured using Microplate Manager at a wavelength of 450 nm.

Total RNA was isolated from the intestinal tissue using TRIzol reagent (Invitrogen, Carlsbad, CA), and cDNA was synthesized using the RevertAid First Strand cDNA Synthesis Kit (Thermo Scientific, Wilmington, DE) according to the manufacturer’s instructions. The mRNA expression of cytokines (TNF-α, IFN-γ, IL-10), heme oxygenase-1 (Hmox-1) and the tight junction (TJ) gene zonula occludens 1 (ZO-1) were quantified using the 7500 Fast Real-Time PCR System (Applied Biosystems, Foster City, CA) and SYBR *Premix Ex Taq* II (Takara, Japan). The expression levels were normalized to those of β-actin. The sequences of the primers used are shown in Table 1.

**Table 1:** Primers used for the inflammatory cytokines and ZO-1.

Name	Sequence
mTNF-α forward primer	5’-cgtggaactggcagaaga-3’
mTNF-α reverse primer	5’-acagaagagcgtggtggc-3’
mIL-10 forward primer	5’-aataagagcaaggcagtggag-3’
mIL-10 reverse primer	5’-tgtatgcttctatgcagttgatga-3’
mIFN-γ forward primer	5’-actaccttcttcagcaacagcaa-3’
mIFN-γ reverse primer	5’-ctggtggaccactcggatga-3’
mZO-1 forward primer	5’-gaccaatagctgatgttgccagag-3’
mZO-1 reverse primer	5’-tatgaaggcgaatgatgccaga-3’
mHmox-1 forward primer	5’-ctggagatgacacctgaggtcaa-3’
mHmox-1 reverse primer	5’-ctgacgaagtgacgccatctg-3’
mβ-actin forward primer	5’-tttccagccttccttctt-3’
mβ-actin reverse primer	5’-gtctttacggatgtcaacg-3’

### Detection and quantitative analysis of engrafted hMSCs in the mouse intestine

The biological samples were subjected DNA extraction and PCR analysis to quantify the numbers of human cells in the recipient mice. Genomic DNA was prepared from intestinal tissues using a QIAamp DNA Mini Kit (Qiagen, Valencia, CA) for PCR analysis. The human β-globin gene and the endogenous mouse receptor-associated protein at the synapse (RAPSYN) gene were amplified using Premix Ex Taq (Probe qPCR) (Takara, Japan). For the human β-globin, the forward primer was 5’-GTGCACCTGACTCCTGAGGAGA-3’, the reverse primer was 5’-CCTTGATACCAACCTGCCCAGG-3’ and the probe, which was labeled with a fluorescent reporter and quencher, was 5’-FAM-AAGGTGAACGTGGATGAAGTTGGTGG-TAMRA-3’. For mouse RAPSYN, the forward primer was 5’-CCTTAGCCAATTGGAGAACA-3’, the reverse primer was 5’-TTGGCCAGTTTAAAACCCAT-3’ and the probe was 5’-FAM-TATCTGACCCACCCATCCTGC-TAMRA-3’.

### Intestinal morphology

#### HE staining

Paraffin-embedded intestinal tissues were sectioned at a thickness of 5 μm and stained with hematoxylin and eosin (H&E). The histopathology of this tissue was evaluated under a light microscope.

#### Immunohistochemistry

The paraffin-embedded intestinal tissue sections were used for immunohistochemistry. Human β2-microglobin, PCNA and mouse ZO-1 were detected using rabbit anti-human β2-microglobin (ab175031, Abcam, UK) (1:200), rabbit anti-PCNA (ab2426, Abcam, UK) (1:200) and anti-ZO-1 antibodies (ab187012, Abcam, UK) (1:500), respectively; then, the sections were immunostained with goat anti-rabbit IgG conjugated to HRP using a DAB kit (ZhongShan Goldenbridge, China).

For terminal deoxynucleotidyl transferase-mediated nick-end labeling (TUNEL) staining of the intestinal tissue, we used the Dead End Fluorometric TUNEL System (Promega, Madison, WI) according to the manufacturer's instructions.

The integrated OD (IOD) of ZO-1 expression was measured and analyzed using an image analysis system (CMIAS-Ⅱ, Beijing University of Aeronautics and Astronautics, China) and was normalized to the negatively stained control. The IOD was quantified as the density (mean) × area of the positively stained objects, representing all of the positive cells in the given field. Cell number quantification of the immunostained tissue sections was performed on 5 random 400× images per group.

### Statistical analysis

The values are presented as means ± SD. One-way ANOVA followed by Dunnett’s post hoc test was used to compare the means from two or more experimental groups. The differences between the groups were considered to be significant at p < 0.05.

## Results

### HGF gene transduction enhances the homing of hMSCs in the intestine of RIII mice

**Fig 1:**
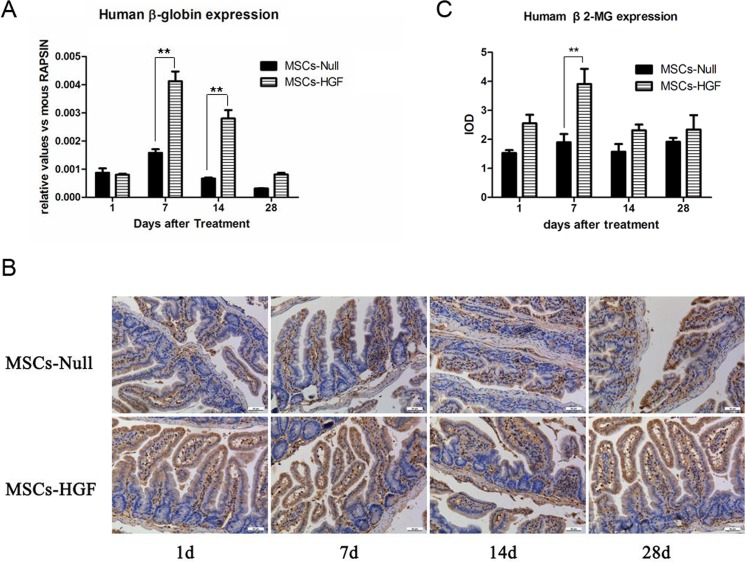
Engraftment of injected hMSCs into the intestine of RIII mouse model. To determine the engraftment of hMSCs into the intestine of RIII mice and compare the efficiency between Ad-HGF modified MSCs (MSCs-HGF) and Ad-Null modified MSCs (MSCs-Null), the expression of human β-globin gene and β2-microglobin in radiation-injured intestinal tissue were detected by quantitative PCR (qPCR) and immunostaining, respectively. At 1, 7, 14 and 28 days post-transplantation, the expression of human β-globin gene in MSCs-Null and MSCs-HGF treated RIII mice were analyzed by qPCR and normalized to the mouse RAPSYN gene (A). To further confirm the engraftment of MSCs into the injured intestinal tissue, the expression of β2-microglobin was detected in MSCs-Null and MSCs-HGF treated RIII mice (B), and were quantitatively analyzed based on IOD value (C). The results are presented as the means ± SD. ** p < 0.01 vs. the MSCs-Null group at the corresponding time point.

To quantitatively analyze the homing of hMSCs in the intestine of RIII mice, we evaluated the expression of the human β-globin gene in radiation-injured intestinal tissue via quantitative PCR at different time points after MSCs therapy. The levels of human β-globin expression in the MSCs-HGF group were higher than those in the MSCs-Null group (*p*< 0.01) (Fig 1A). Furthermore, β2-microglobin staining and IOD analysis showed that human MSCs (hMSCs) homed to sites of the lamina propria surrounding the small intestinal epithelial crypts (Fig 1B and 1C). These results indicated that HGF gene modification enhanced the homing of these cells to radiation injured intestine.

### hMSC treatment reduces intestinal damage and restores intestinal integrity in RIII mice

**Fig 2:**
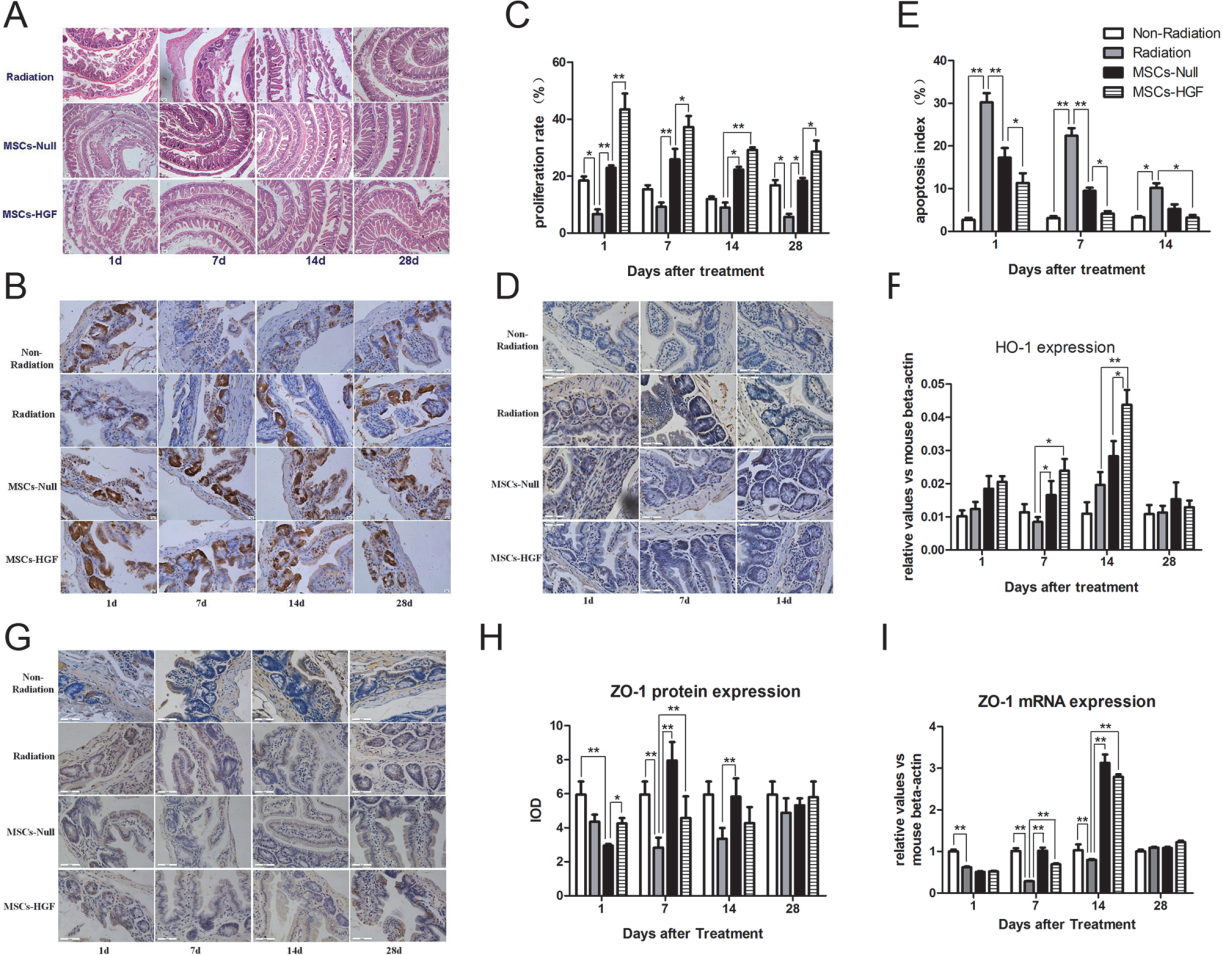
The infusion of MSCs-Null or MSCs-HGF restores the integrity of the intestine in an RIII mouse model. Small intestinal tissue samples from radiation, MSCs-Null and MSCs-HGF groups were obtained at 1, 7, 14 and 28 days post-treatment. The morphological characteristics of small intestinal integrity were detected in radiation by hematoxylin-eosin (HE) staining (A). The proliferation of intestinal epithelial cells was tested by proliferating cell nuclear antigen (PCNA) staining. The representative images were shown in B and proliferation rate was quantified (C). The apoptosis in the crypt and villus of the intestine was analyzed by terminal deoxynucleotidyl transferase dUTP nick end labeling (TUNEL) (D), and the apoptotic index was shown in E. ZO-1, an important scaffold protein in TJs, was detected by immunostaining at protein level (G, H) and q-PCR at mRNA level (I) in the intestine. The expression of heme oxygenase-1 (HO-1), an enzyme that degrades heme and plays an important role in the cellular protection against oxidative stress and apoptosis, were also analyzed by q-PCR (F). All the data were presented as the means ± SD. Comparisons between groups were analyzed via one-way ANOVA followed by Dunnett’s post hoc test. *p<0.05, **p<0.01.

Histological analysis showed that radiation could induce epithelial atrophy, which is characterized by the loss of intestinal structural integrity, at day 1 post-radiation and recovered within 28 days. Then, the effects of MSCs-Null or MSCs-HGF treatment on small intestinal integrity (structure) were evaluated. Both in MSCs-Null and MSCs-HGF treated group, the structural integrity was restored at day 7 post-treatment, indicating that hMSCs treatment stimulates the structural re-epithelization of the small intestine in this mouse RIII model. The level of intestinal epithelial hypertrophy progressively decreased in RIII mice receiving MSCs treatment (Fig 2A).

To clarify the mechanisms of the protective roles of MSCs-Null and MSCs-HGF in this RIII model, we analyzed the proliferation and apoptosis rates of intestinal epithelial cells. PCNA staining showed that MSCs-HGF or MSCs-Null significantly induced the proliferation of intestinal epithelial cells compared with the saline control injection in RIII mice (Fig 2B and 2C). In MSCs-HGF group, the maximal increase was observed at 1 day after treatment, and then followed by a slight decline. Apoptotic cells in the small intestinal tissue were stained with TUNEL. The results showed that most of the apoptotic cells located in the crypt and villus of the intestine. In saline treated mice, the increase of apoptotic cells reached peak at day 1, and could be detected until 14 days after treatment. However, the rate of apoptosis was significantly decreased in the MSCs-HGF- and MSCs-Null-treated mice from day 1 to 14 (Fig 2D and 2E). Heme oxygenase-1 (HO-1), an enzyme that degrades heme, plays an important role in the cellular protection against oxidative stress and apoptosis. qPCR revealed that HO-1 expression was up-regulated by MSCs-Null or MSCs-HGF, especially at 7 and 14 days post-treatment (Fig 2F).

The intestinal epithelial TJ is considered as an index of epithelial cell injury. And, ZO-1 is an important scaffold protein in TJs [17, 18]. We detected both ZO-1 protein (Fig 2G and 2H) and mRNA expression (Fig 2I) in the intestines of RIII mice. Radiation disrupted ZO-1 expression, which maximally decreased at 7 days after radiation. The administration of MSCs-Null and MSCs-HGF blocked this decrease and stimulated the expression of ZO-1, which peaked at 14 days after radiation.

### MSCs-HGF therapy reduces the inflammatory responses in RIII mice

**Fig 3:**
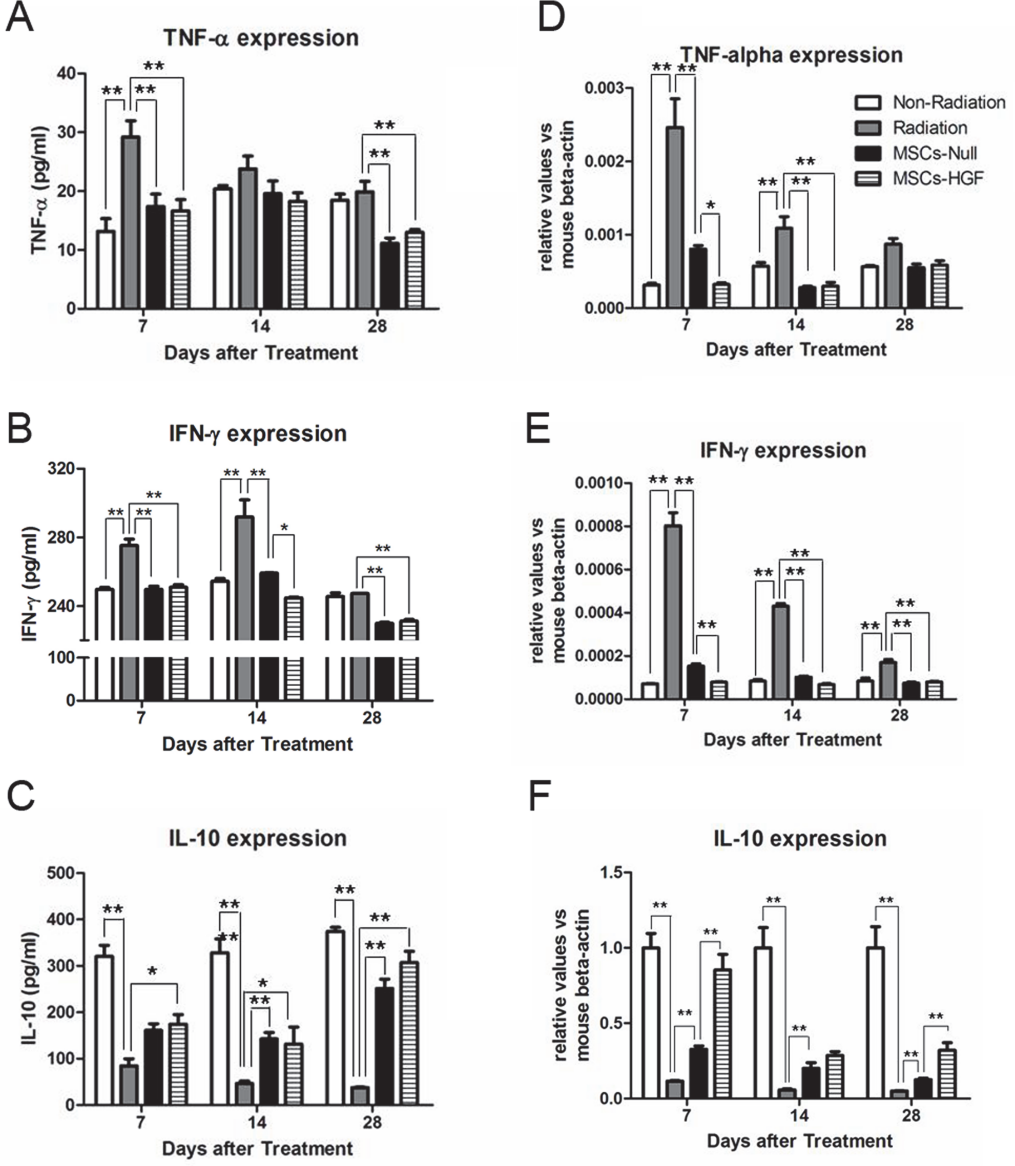
The expression levels of pro-inflammatory and anti-inflammatory cytokines in peripheral blood and the intestinal tissue. The immunomodulatory activities of MSCs-Null and MSCs-HGF were evaluated by detected the expression and secretion of pro-inflammatory cytokines TNF-α and IFN-γ, and the anti-inflammatory cytokine IL-10. Peripheral blood samples were collected from RIII mice at 7, 14 and 28 days after treatments. The expression levels of the pro-inflammatory cytokines TNF-α (A) and IFN-γ (B) and the anti-inflammatory cytokine IL-10 (C) were measured via ELISA. On the other hand, total RNA was extracted from the intestinal tissue, and the mRNA expression levels of TNF-α (D), IFN-γ (E) and IL-10 (F) were measured by real-time RT-PCR, and normalized to the expression of mouse β-actin. All the data are presented as the means ± SD. Comparisons between groups were analyzed via one-way ANOVA followed by Dunnett’s post hoc test. * p<0.05; ** p<0.01.

The immunomodulatory activities of MSCs-null and MSCs-HGF were evaluated in the RIII model. The protein levels of IL-10, TNF-α and IFN-γ in peripheral blood were measured via ELISA at 7, 14 and 28 days post-radiation. As shown in Fig 3A and 3B, MSCs treatment reduced the serum levels of inflammatory cytokines (TNF-α, and IFN-γ) in RIII mice. Moreover, the level of the anti-inflammatory/regulatory cytokine IL-10 in the MSCs-treated groups was significantly higher than in the saline control group (Fig 3C). Furthermore, the mRNA expression levels of TNF-α, IFN-γ and IL-10 in the local small intestinal tissue were detected by real-time RT-PCR. Compared with the radiation group, the MSCs-Null and MSCs-HGF groups displayed reduced mRNA expression levels of TNF-α and IFN-γ (Fig 3D and 3E) and increased expression levels of IL-10 (Fig 3F).

### MSCs-HGF regulates MLN and PP lymphocytes in RIII mice

**Fig 4:**
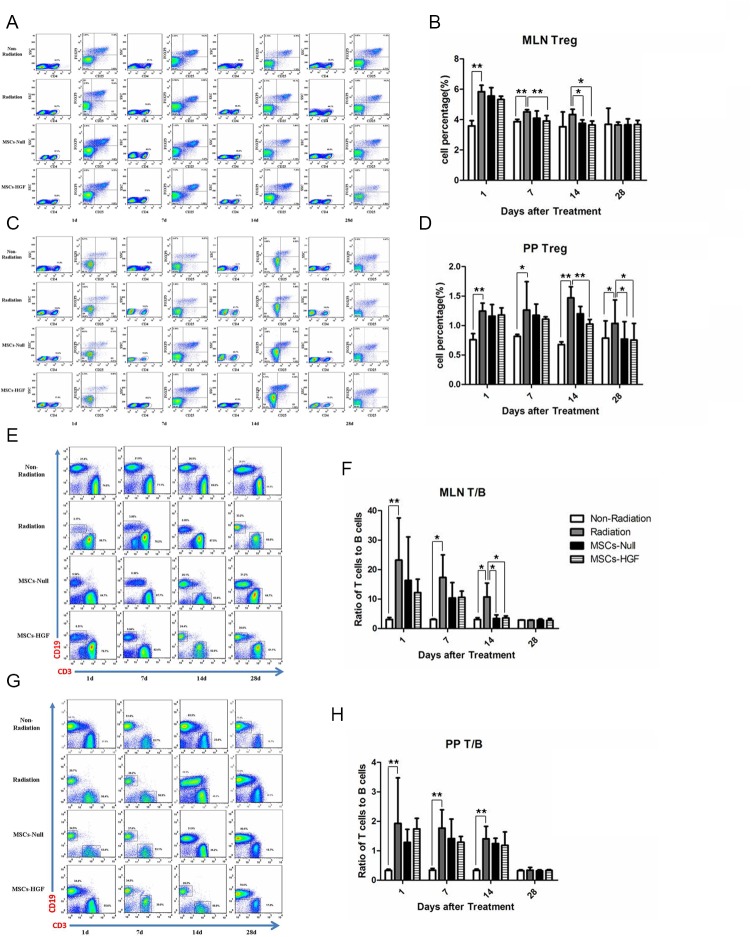
Immunophenotypic changes in MLN and PP lymphocytes after radiation. At 1, 7, 14 and 28 days after treatments, MLNs and PPs were carefully collected, and were mechanically dissociated in phosphate-buffered saline (PBS) containing 3% fetal calf serum (FCS). Lymphocytes were isolated from the MLNs and PPs and were labeled with fluorescent antibodies, and immunophenotypic differences were detected via FACS. Tregs in the MLNs (A, B) and PPs (C, D) were labeled with the regulatory T cell staining kit. The T and B lymphocytes were detected after labeled by CD3 FITC, anti-mouse CD19 PerCP-Cy5.5, anti-mouse CD4 PE, anti-mouse CD8b APC or isotype control. The ratio of T cells/B cells in the MLNs (E, F) and PPs (G, H) are shown. Representative images of FACS are shown. The results are presented as the means ± SD. Comparisons between groups were analyzed via one-way ANOVA followed by Dunnett’s post hoc test. *p<0.05; **p<0.01.

Lymphocytes from the MLN and PP in the small intestine of radiated and untreated, MSCs-Null-treated or MSCs-HGF-treated mice were stained with antibodies and analyzed via FACS. As shown in Fig 4, radiation induced an increase in CD4^+^CD25^+^Foxp3^+^ Treg cells, which persisted for approximately 14 days. Treatment with MSCs-Null or MSCs-HGF significantly reduced the percentage of CD4^+^CD25^+^Foxp3^+^ Treg cells in RIII mice (Fig 4A–4D). The radiation-induced increase in the T cell to B cell ratios in the MLN and PP were also alleviated by MSCs-Null or MSCs-HGF treatment (Fig 4E–4H).

## Discussion

The major pathological change caused by RIII is architectural disorganization, including inflammatory mononuclear cell infiltration, villitis, desquamation, eosinophilic necrosis, and reduced mucosal thickness, crypt height, and villous height [19]. Effective therapies may accelerate the structural regeneration of the small intestine and attenuate inflammation in RIII.

Both stem cell and gene therapy are promising approaches for the replenishment of the radiation injury-induced depletion of stem cell compartments. Studies have shown that MSCs display the capacity to engraft into the enteric mucosa in the radiated intestinal tissue of mice [20]. In this study, UC-MSCs transduced with Ad-HGF or Ad-Null was injected intravenously into RIII mice. The distribution of MSCs in the injured tissues was determined via real-time PCR. The human β-globin gene expression level was higher and was sustained for longer in the MSCs-HGF group than in the MSCs-Null group. It was demonstrated that MSCs characteristically home to radiation-injured intestinal tissue and that HGF gene transduction enhances the transient homing of hMSCs in the intestines of RIII mice. This effect was independent of the proliferative characteristics of MSCs-HGF (S1 Fig).

MSCs-based gene therapy may exert beneficial effects on RIII therapy. Yang et al. have demonstrated that bone marrow-derived MSCs and the overexpression of human manganese superoxide dismutase ameliorated RIII [21]. MSCs modulate inflammatory responses and tissue regeneration via multiple mechanisms. Recent studies have shown that MSCs ameliorated dextran sodium sulfate induced colitis via a local anti-inflammatory action [22]. The secretion of a broad range of bioactive molecules that alter the tissue microenvironment is currently believed to serve as the primary mechanism by which MSCs exert their therapeutic effects. The transplanted MSCs might, as a principal mechanism, export their inherent trophic effects to unorthodox sites [23]. MSCs treatment results in the enhanced regeneration of injured cells, the stimulation of the proliferation and differentiation of endogenous tissue progenitors, and a decrease in inflammatory and immune reactions [24, 25]. Moreover, MSCs escape allogeneic rejection in human and animal models because they do not express HLA-DR. The specific immune reactions against human-derived MSCs were not detected in the mice used in this study, suggesting that hMSCs exhibit low immunogenicity (S2 Fig).

Many cytokines are involved in the repair of radiation-injured tissue. HGF, a growth factor that performs multiple functions, is involved in not only liver regeneration but also the repair of other tissues. Studies showed that HGF modulates the proliferation and migration of intestinal epithelial cells, leading to the acceleration of intestinal mucosal repair and serving as a critical regulator of intestinal wound healing [12, 14]. The systemic administration of recombinant human HGF protein ameliorated experimental colitis. However, an increase in the serum HGF concentrations may induce systemic adverse effects. In this study, we measured the expression level of human and mouse HGF in the circulation. The results showed that the expression level of HGF in the circulation increased after radiation and MSCs treatments, but at a low level (S3 Fig and S4 Fig). However, MSCs-based HGF gene therapy increases HGF expression locally in damaged tissues in RIII. Compared to the MSCs-Null group, HGF gene modification significantly enhanced the proliferation of intestinal epithelial cells. Additionally, the immunomodulatory activities of MSCs, such as the suppression of T-cell proliferation and the secretion of inflammatory factors, were detected. The expression levels of the inflammatory cytokines TNF-α and IFN-γ and the anti-inflammatory cytokine IL-10 in the peripheral blood were similar between the control and MSCs-Null groups, whereas in the MSCs-HGF group, IL-10 expression was significantly increased and TNF-α and IFN-γ expression was significantly decreased in local tissues at 7 days after treatment. We deduced that this HGF-enhanced anti-inflammatory effect might depend on the engraftment of MSCs into the radiation-injured intestinal tissue.

Radiation causes inflammation and dysregulation of immune homeostasis. Intestinal CD4+T cells may influence the effectiveness of radiotherapy and impede tissue repair in RIII. Thymus-derived natural T regulatory cells (CD4+CD25+FoxP3+ Tregs), the predominant specialized cell type that maintains immune tolerance, play an important role in immune regulation in the intestine. The expression pattern of Tregs is critical for their function [26]. Lymphocytes involved in the intestinal immune response are located in organized immune-inductive sites of the gut-associated lymphoid tissues (GALT), such as PPs, and in draining gut MLNs [27]. In the present study, the ratio of CD4+CD25+FoxP3+ Tregs to CD4+ cells in the MLN and PP were increased after radiation, and MSCs-Null or MSCs-HGF treatment partially ameliorated this up-regulation. Rradiation damaged the lymphoid tissues by decreasing the absolute number of lymphocytes. A study by Linard [28] showed that the absolute number of CD4-T and CD8-T cells in the MLNs progressively decreased and that the CD4-T and CD8-T populations may be differentially sensitive to radiation. We observed that the increased ratio of T/B cells and CD4 T/CD8 T cells (data not shown) coincided with the decrease in the MLN volume after abdominal radiation. This result demonstrated that B cells were more sensitive to radiation than T cells and CD8-T cells were more sensitive to radiation than CD4-T cells. The mechanism underlying immune regulation effect of MSCs maybe include secretion of inhibiting factors responsible for lymphocyte activation and expression of cell surface molecules [29].

Previously, we found that HGF gene modification exerts beneficial effects on cell transplantation and tissue repair [18]. Furthermore, MSCs-based HGF gene therapy reduces inflammation and inhibits lung fibrosis in a radiation-induced lung injury model [30]. According to previous studies on MSCs and HGF, both direct effects of homing and restoration and secondary effects of secretion of growth factors were included in the effects of HGF-modified MSCs on RIII.

The low number of human-derived cells that were implanted into the intestinal mucosa indicates that the replacement of epithelial cell loss via trans-differentiation is unlikely to represent the principal therapeutic mechanism [7]. Nevertheless, MSC treatment enhanced radiation-induced epithelial crypt cell proliferation and reduced radiation-induced epithelial apoptosis in the intestine. Moreover, the anti-apoptotic effect of MSCs-HGF was significantly greater than that of MSCs-Null, suggesting the synergistic function of MSCs and HGF.

Epithelial barrier function is largely determined by a multiprotein complex located at the most apical portion of the lateral membrane, which is referred to as a TJ. ZO-1 is a scaffolding protein that plays a pivotal role in the formation of TJs [17, 31]. A variety of inflammatory mediators, such as ROS and inflammatory cytokines including TNF-α and IFN-γ, disrupt the TJ barrier and alter the localization and phosphorylation status of ZO-1. In the present study, we observed that radiation decreased the expression of ZO-1 based on RT-PCR and immunostaining. The expression of ZO-1, which is predominantly located along the apical surface of intestinal villi, was reduced, indicating that the localization of ZO-1 to the TJs was disturbed. The administration of MSCs-Null or MSCs-HGF alleviated the radiation-induced decrease in ZO-1 expression, and this effect may play an important role in the maintenance of epithelial barrier function.

## Conclusion

In conclusion, the application of MSCs and HGF-modified MSCs protects the intestine from radiation-induced injury, including improving intestinal histopathology, reducing local and systemic inflammation, and increasing the proliferation and decreasing the apoptosis of intestinal epithelial cells. Furthermore, HGF gene modification enhances the homing of MSCs to radiation-injured intestinal tissue, which contributes to the improvement of tissue repair and the modulation of inflammation in the local intestine. Therefore, HGF-modified MSCs may represent an effective therapeutic strategy for RIII.

## Supporting Information

S1 Fig HGF expression does not affect the proliferation of MSCs. Proliferation of MSCs was determined by using Dye eFluor 670, representative results are shown.
(TIF)

S2 Fig Anti-Human MSC reaction in mice serum. The antibody against human MSCs was determined by using ELISA technique. Results are shown as the mean ± SD.
(TIF)

S3 Fig Human HGF Expression in mice serum. The expression of human HGF was determined by using ELISA technique. Results are shown as the mean ± SD. ** p < 0.01, vs MSCs-Null group at the same time point.
(TIF)

S4 Fig Mouse HGF Expression in mice serum. The expression of mouse HGF was determined by using ELISA technique. Results are shown as the mean ± SD. *p < 0.05, ** p < 0.01.
(TIF)

S1 Methods The Effect of Ad-HGF transduction on proliferation of MSCs, detected by Cell Proliferation Dye eFluor 670 at 24h, 48h or 96h post-transduction. The human HGF and mouse HGF expression in serum was measured by ELISA at 1, 7, 14 and 28d ays post-radiation. And, the antibody against MSCs also examined at 7 and 14 days post-radiation by using ELISA.
(DOCX)

## References

[1] Monti P, Wysocki J, van der Meeren A, Griffiths NM. The contribution of radiation-induced injury to the gastrointestinal tract in the development of multi-organ dysfunction syndrome or failure. BJR supplement / BIR. 2005;27:89–94.

[2] Kudo K, Liu Y, Takahashi K, Tarusawa K, Osanai M, Hu DL, et al Transplantation of mesenchymal stem cells to prevent radiation-induced intestinal injury in mice. Journal of radiation research. 2010;51(1):73–9. 19851042 10.1269/jrr.09091

[3] Rodrigo SF, van Ramshorst J, Hoogslag GE, Boden H, Velders MA, Cannegieter SC, et al Intramyocardial injection of autologous bone marrow-derived ex vivo expanded mesenchymal stem cells in acute myocardial infarction patients is feasible and safe up to 5 years of follow-up. Journal of cardiovascular translational research. 2013;6(5):816–25. 10.1007/s12265-013-9507-7 23982478 PMC3790917

[4] Galipeau J. The mesenchymal stromal cells dilemma—does a negative phase III trial of random donor mesenchymal stromal cells in steroid-resistant graft-versus-host disease represent a death knell or a bump in the road? Cytotherapy. 2013;15(1):2–8. 10.1016/j.jcyt.2012.10.002 23260081

[5] Hernigou P, Pariat J, Queinnec S, Homma Y, Flouzat Lachaniette CH, Chevallier N, et al Supercharging irradiated allografts with mesenchymal stem cells improves acetabular bone grafting in revision arthroplasty. International orthopaedics. 2014;38(9):1913–21. 10.1007/s00264-014-2285-2 24509980

[6] Reagan MR, Seib FP, McMillin DW, Sage EK, Mitsiades CS, Janes SM, et al Stem Cell Implants for Cancer Therapy: TRAIL-Expressing Mesenchymal Stem Cells Target Cancer Cells In Situ. Journal of breast cancer. 2012;15(3):273–82. 10.4048/jbc.2012.15.3.273 23091539 PMC3468780

[7] Semont A, Mouiseddine M, Francois A, Demarquay C, Mathieu N, Chapel A, et al Mesenchymal stem cells improve small intestinal integrity through regulation of endogenous epithelial cell homeostasis. Cell death and differentiation. 2010;17(6):952–61. 10.1038/cdd.2009.187 20019749

[8] Garcia-Olmo D, Garcia-Arranz M, Herreros D, Pascual I, Peiro C, Rodriguez-Montes JA. A phase I clinical trial of the treatment of Crohn's fistula by adipose mesenchymal stem cell transplantation. Diseases of the colon and rectum. 2005;48(7):1416–23. 15933795 10.1007/s10350-005-0052-6

[9] Selleri S, Dieng MM, Nicoletti S, Louis I, Beausejour C, Le Deist F, et al Cord-blood-derived mesenchymal stromal cells downmodulate CD4+ T-cell activation by inducing IL-10-producing Th1 cells. Stem cells and development. 2013;22(7):1063–75. 10.1089/scd.2012.0315 23167734 PMC3608091

[10] Luz-Crawford P, Kurte M, Bravo-Alegria J, Contreras R, Nova-Lamperti E, Tejedor G, et al Mesenchymal stem cells generate a CD4+CD25+Foxp3+ regulatory T cell population during the differentiation process of Th1 and Th17 cells. Stem cell research & therapy. 2013;4(3):65. 23734780 10.1186/scrt216 PMC3706898

[11] Matsumoto K, Nakamura T. Hepatocyte growth factor (HGF) as a tissue organizer for organogenesis and regeneration. Biochemical and biophysical research communications. 1997;239(3):639–44. 9367820 10.1006/bbrc.1997.7517

[12] Setoyama H, Ido A, Numata M, Moriuchi A, Yamaji N, Tamai T, et al Repeated enemas with hepatocyte growth factor selectively stimulate epithelial cell proliferation of injured mucosa in rats with experimental colitis. Life sciences. 2011;89(7–8):269–75. 21763320 10.1016/j.lfs.2011.06.019

[13] Kanayama M, Takahara T, Yata Y, Xue F, Shinno E, Nonome K, et al Hepatocyte growth factor promotes colonic epithelial regeneration via Akt signaling. American journal of physiology Gastrointestinal and liver physiology. 2007;293(1):G230–9. 17412827 10.1152/ajpgi.00068.2007

[14] Ido A, Numata M, Kodama M, Tsubouchi H. Mucosal repair and growth factors: recombinant human hepatocyte growth factor as an innovative therapy for inflammatory bowel disease. Journal of gastroenterology. 2005;40(10):925–31. 16261428 10.1007/s00535-005-1705-x

[15] Lu LL, Liu YJ, Yang SG, Zhao QJ, Wang X, Gong W, et al Isolation and characterization of human umbilical cord mesenchymal stem cells with hematopoiesis-supportive function and other potentials. Haematologica. 2006;91(8):1017–26. 16870554

[16] Hu ZB, Wu CT, Wang H, Zhang QW, Wang L, Wang RL, et al A simplified system for generating oncolytic adenovirus vector carrying one or two transgenes. Cancer gene therapy. 2008;15(3):173–82. 18157145 10.1038/sj.cgt.7701105

[17] Hamada K, Shitara Y, Sekine S, Horie T. Zonula Occludens-1 alterations and enhanced intestinal permeability in methotrexate-treated rats. Cancer chemotherapy and pharmacology. 2010;66(6):1031–8. 10.1007/s00280-010-1253-9 20119715

[18] Bian L, Guo ZK, Wang HX, Wang JS, Wang H, Li QF, et al In vitro and in vivo immunosuppressive characteristics of hepatocyte growth factor-modified murine mesenchymal stem cells. In Vivo. 2009;23(1):21–7. 19368120

[19] Onal C, Kayaselcuk F, Topkan E, Yavuz M, Bacanli D, Yavuz A. Protective effects of melatonin and octreotide against radiation-induced intestinal injury. Digestive diseases and sciences. 2011;56(2):359–67. 10.1007/s10620-010-1322-2 20652743

[20] Zhang J, Gong JF, Zhang W, Zhu WM, Li JS. Effects of transplanted bone marrow mesenchymal stem cells on the irradiated intestine of mice. Journal of biomedical science. 2008;15(5):585–94. 10.1007/s11373-008-9256-9 18763056

[21] Yang C, Chen HX, Zhou Y, Liu MX, Wang Y, Wang JX, et al Manganese superoxide dismutase gene therapy protects against irradiation- induced intestinal injury. Current gene therapy. 2013;13(5):305–14. 24060314 10.2174/15665232113136660027

[22] Tanaka F, Tominaga K, Ochi M, Tanigawa T, Watanabe T, Fujiwara Y, et al Exogenous administration of mesenchymal stem cells ameliorates dextran sulfate sodium-induced colitis via anti-inflammatory action in damaged tissue in rats. Life sciences. 2008;83(23–24):771–9. 10.1016/j.lfs.2008.09.018 18950645

[23] Caplan AI, Dennis JE. Mesenchymal stem cells as trophic mediators. Journal of cellular biochemistry. 2006;98(5):1076–84. 16619257 10.1002/jcb.20886

[24] Phinney DG, Prockop DJ. Concise review: mesenchymal stem/multipotent stromal cells: the state of transdifferentiation and modes of tissue repair—current views. Stem Cells. 2007;25(11):2896–902. 17901396 10.1634/stemcells.2007-0637

[25] Lange C, Brunswig-Spickenheier B, Cappallo-Obermann H, Eggert K, Gehling UM, Rudolph C, et al Radiation rescue: mesenchymal stromal cells protect from lethal irradiation. PloS one. 2011;6(1):e14486 10.1371/journal.pone.0014486 21245929 PMC3016319

[26] Billiard F, Buard V, Benderitter M, Linard C. Abdominal gamma-radiation induces an accumulation of function-impaired regulatory T cells in the small intestine. International journal of radiation oncology, biology, physics. 2011;80(3):869–76. 10.1016/j.ijrobp.2010.12.041 21345609

[27] Turner JD, Jenkins GR, Hogg KG, Aynsley SA, Paveley RA, Cook PC, et al CD4+CD25+ regulatory cells contribute to the regulation of colonic Th2 granulomatous pathology caused by schistosome infection. PLoS neglected tropical diseases. 2011;5(8):e1269 10.1371/journal.pntd.0001269 21858239 PMC3153428

[28] Linard C, Billiard F, Benderitter M. Intestinal irradiation and fibrosis in a Th1-deficient environment. International journal of radiation oncology, biology, physics. 2012;84(1):266–73. 10.1016/j.ijrobp.2011.11.027 22336200

[29] Madrigal M, Rao KS, Riordan NH. A review of therapeutic effects of mesenchymal stem cell secretions and induction of secretory modification by different culture methods. Journal of translational medicine. 2014;12(1):260 25304688 10.1186/s12967-014-0260-8 PMC4197270

[30] Wang H, Yang YF, Zhao L, Xiao FJ, Zhang QW, Wen ML, et al Hepatocyte growth factor gene-modified mesenchymal stem cells reduce radiation-induced lung injury. Human gene therapy. 2013;24(3):343–53. 10.1089/hum.2012.177 23458413

[31] Xu LF, Xu C, Mao ZQ, Teng X, Ma L, Sun M. Disruption of the F-actin cytoskeleton and monolayer barrier integrity induced by PAF and the protective effect of ITF on intestinal epithelium. Archives of pharmacal research. 2011;34(2):245–51. 10.1007/s12272-011-0210-4 21380808

