# Supplementary figures and images for: HGF Gene Modification in Mesenchymal Stem Cells Reduces Radiation-Induced Intestinal Injury by Modulating Immunity
Source: PLoS One. 2015 May 1;10(5):e0124420. doi: 10.1371/journal.pone.0124420 (PMC4416803; doi:10.1371/journal.pone.0124420)

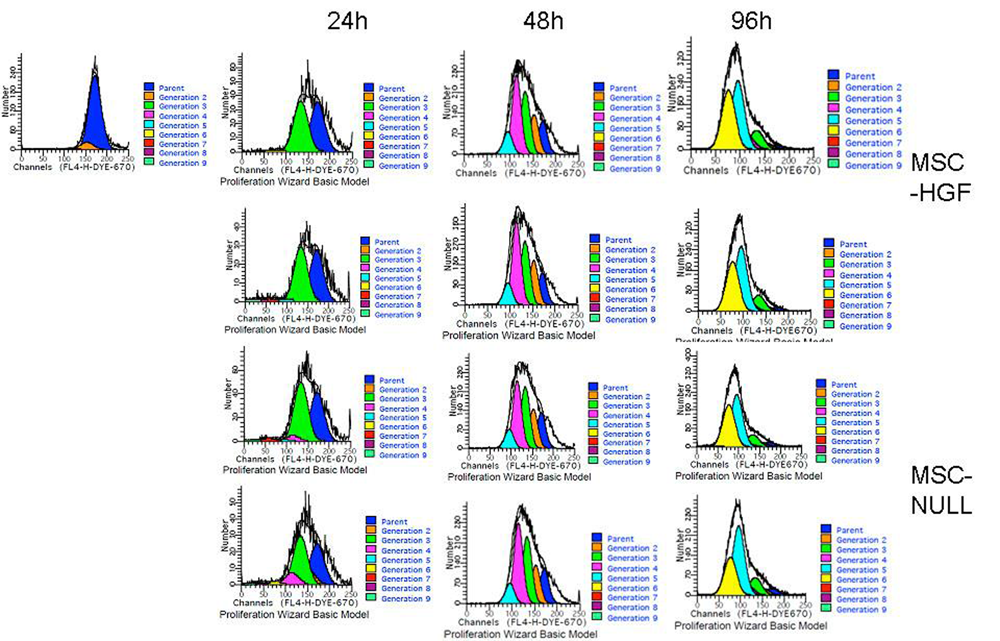

Supplement: S1 Fig — Proliferation of MSCs was determined by using Dye eFluor 670, representative results are shown. (TIF) [file pone.0124420.s001.tif]

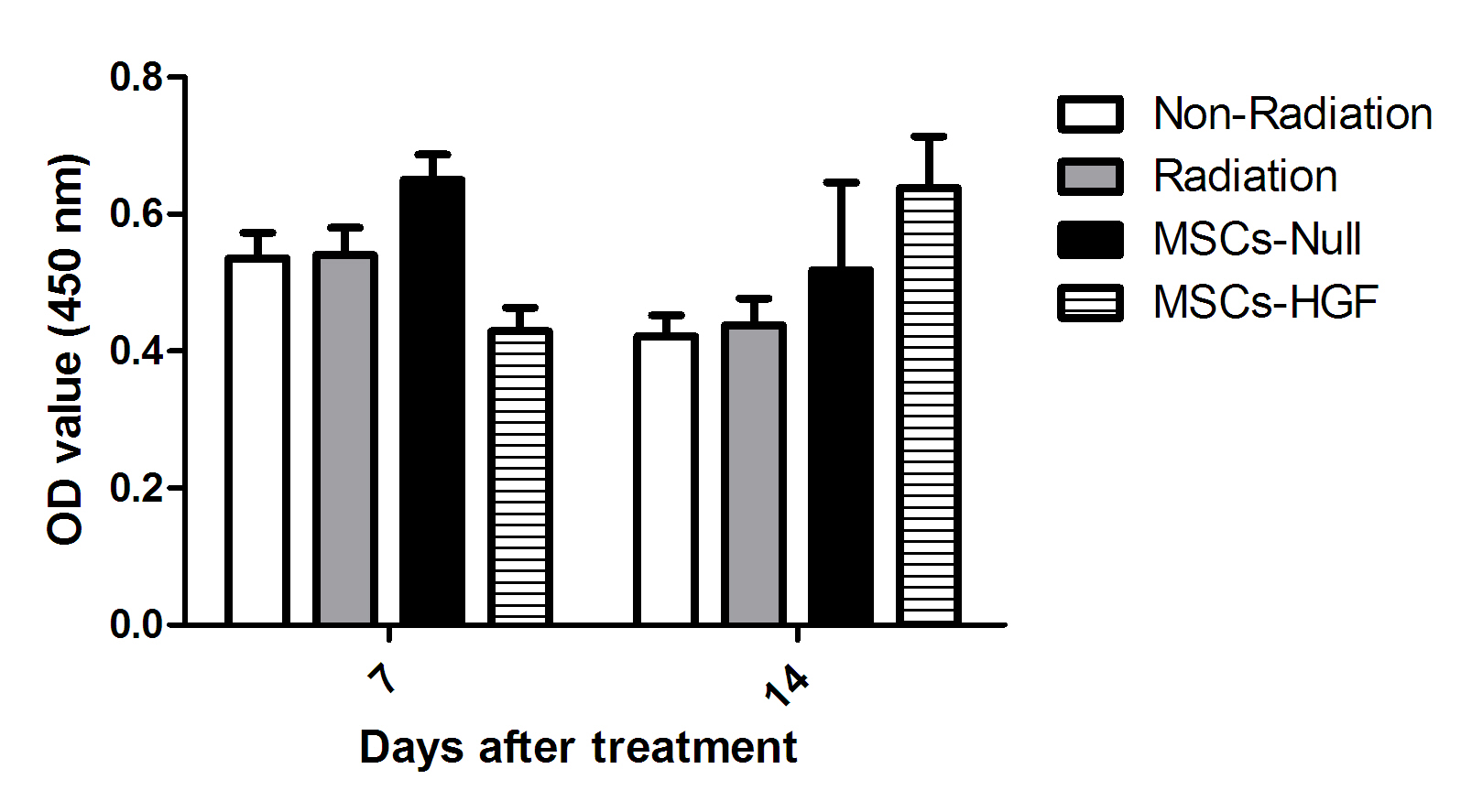

Supplement: S2 Fig — The antibody against human MSCs was determined by using ELISA technique. Results are shown as the mean ± SD. (TIF) [file pone.0124420.s002.tif]

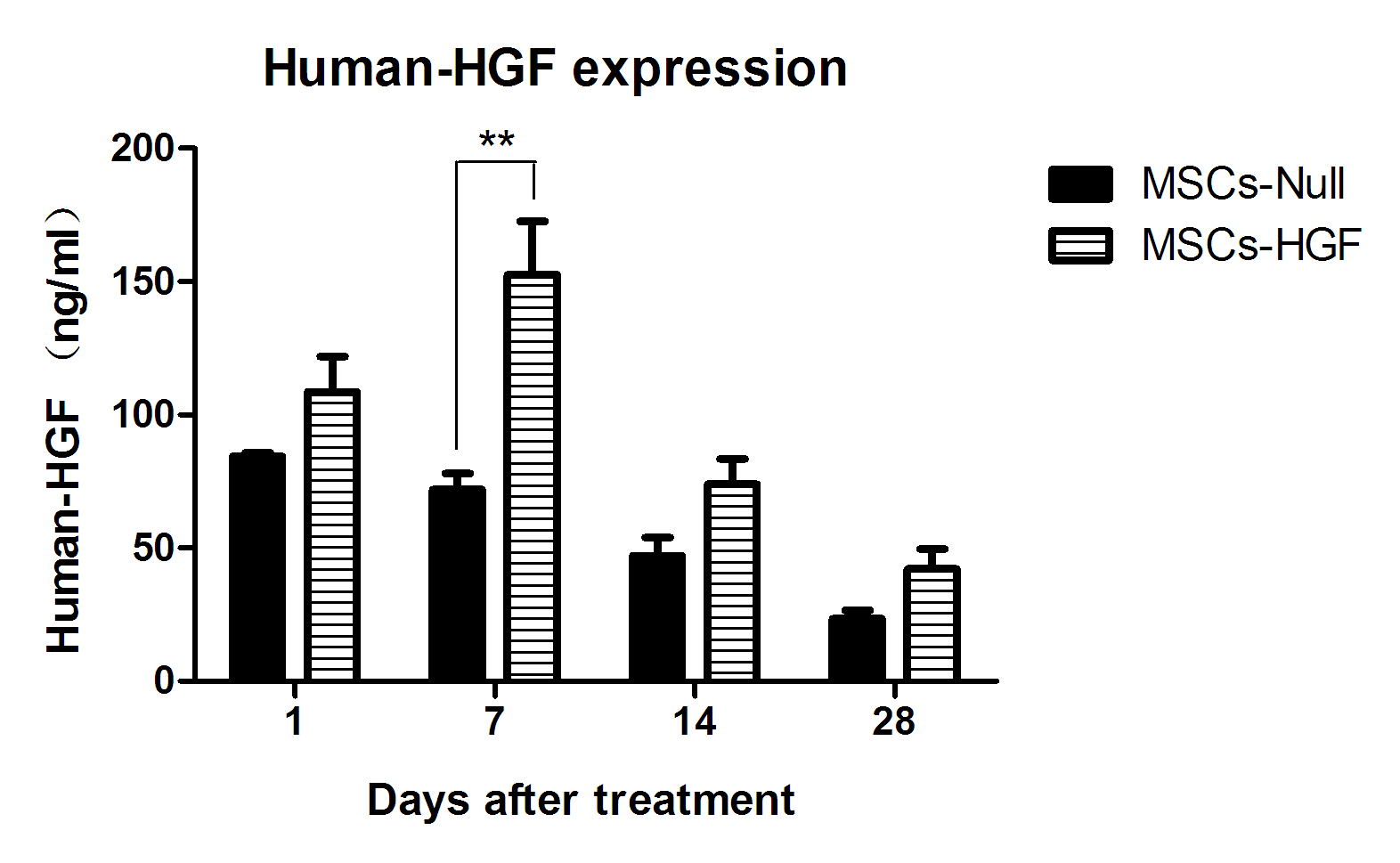

Supplement: S3 Fig — The expression of human HGF was determined by using ELISA technique. Results are shown as the mean ± SD. ** p < 0.01, vs MSCs-Null group at the same time point. (TIF) [file pone.0124420.s003.tif]

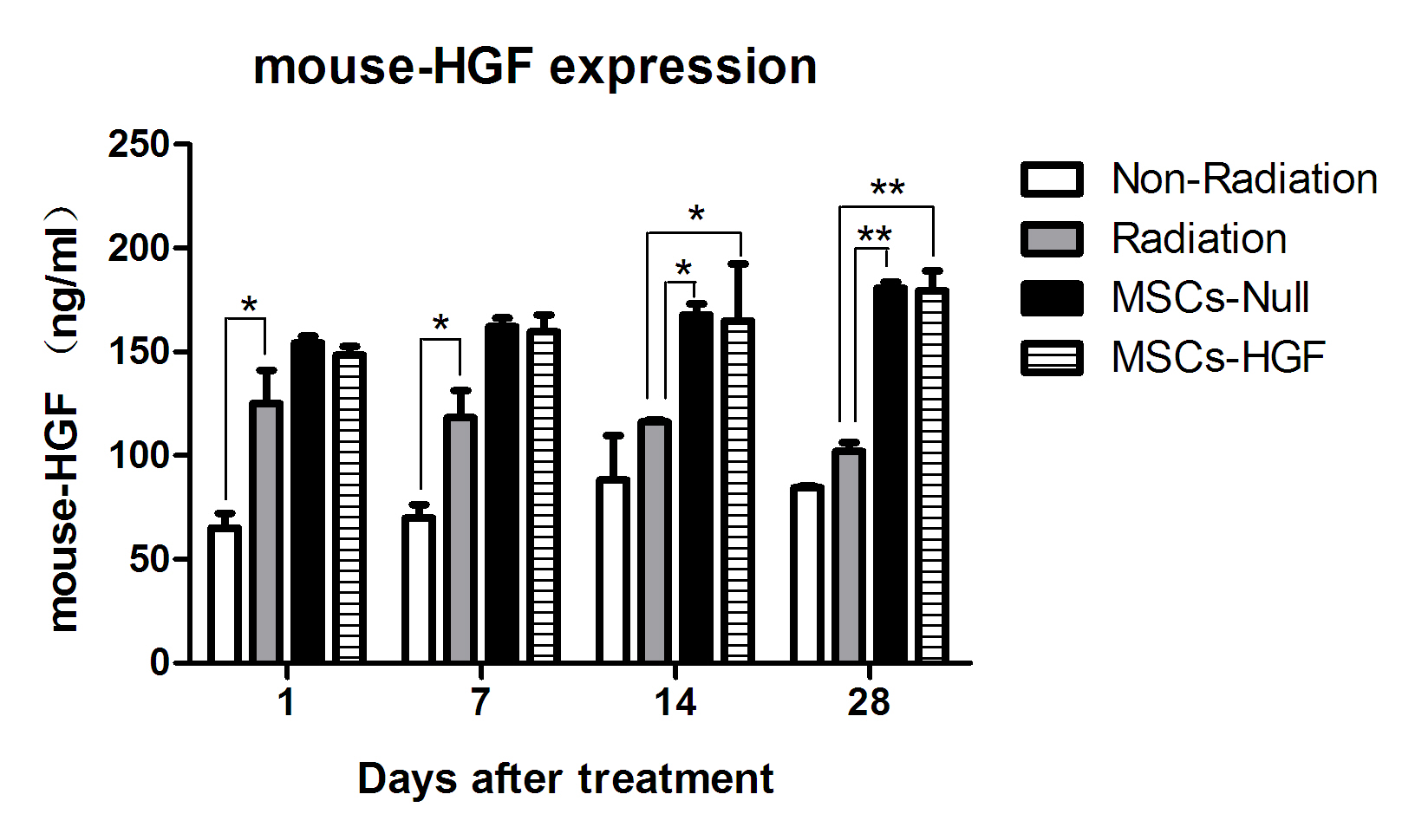

Supplement: S4 Fig — The expression of mouse HGF was determined by using ELISA technique. Results are shown as the mean ± SD. *p < 0.05, ** p < 0.01. (TIF) [file pone.0124420.s004.tif]
