# Supplementary material for: HGF Gene Modification in Mesenchymal Stem Cells Reduces Radiation-Induced Intestinal Injury by Modulating Immunity
Source: PLoS One. 2015 May 1;10(5):e0124420. doi: 10.1371/journal.pone.0124420 (PMC4416803; doi:10.1371/journal.pone.0124420)
Supplement: S1 Methods — The human HGF and mouse HGF expression in serum was measured by ELISA at 1, 7, 14 and 28d ays post-radiation. And, the antibody against MSCs also examined at 7 and 14 days post-radiation by using ELISA. (DOCX) [file pone.0124420.s005.docx]

**Supporting Materials and methods**

***The Effect of Ad-HGF transduction on proliferation of MSCs***

MSCs were labeled by Cell Proliferation Dye eFluor® 670 (Biosciences Pharmingen, San Diego, CA) according to manufacturer's instructions. These cells were transduced with Ad-HGF or Ad-Null at 150MOI and assayed for proliferation at 24 hours，48 hours or 96 hours post-transduction. The position of the parent generation was set based on a cell aliquot after Dye670 labeling and proliferation index was calculated using ModFit software.

***Detection of human HGF and mouse HGF and antibodies against MSCs***

Blood samples were obtained from tail vein at day1, 7, 14 and 28 post radiation. The concentrations of human HGF (R&D Systems, Minneapolis, MN) and mouse HGF (RapidBio, West Hills, CA) were measured with specific Quantikine ELISA kits respectively according to the manufacturer's instructions.

For detection of antibodies against human MSCs, The ELISA plates were coated with human-derived MSCs lysates at 2μg/ml overnight. Then the mice serum of 7 and 14 day post-radiation of four groups were added and incubated for 2 hours. After washed with PBS for three-times, the plates were added HRP-conjugated goat anti-mouse IgG (H+L) (GAM007, Multi Sciences (Lianke) Biotech co., Ltd, HangZhou, China) and incubated for another hour. Then incubated with TMB (E0213, Multi Sciences (Lianke) Biotech co., Ltd, HangZhou, China) for 15 min. The reaction was stopped by incubating with stop solution (E0301, Multi Sciences (Lianke) Biotech co., Ltd, HangZhou, China) for 15 mins. The optical density (OD) value was measured using Microplate Manager 450 at 450-nm wavelength.
